# Community Structure and Succession Regulation of Fungal Consortia in the Lignocellulose-Degrading Process on Natural Biomass

**DOI:** 10.1155/2014/845721

**Published:** 2014-01-19

**Authors:** Baoyu Tian, Chunxiang Wang, Ruirui Lv, Junxiong Zhou, Xin Li, Yi Zheng, Xiangyu Jin, Mengli Wang, Yongxia Ye, Xinyi Huang, Ping Liu

**Affiliations:** Engineering Research Center of Industrial Microbiology of Ministry of Education, and College of Life Sciences, Fujian Normal University, Fuzhou 350108, China

## Abstract

The study aims to investigate fungal community structures and dynamic changes in forest soil lignocellulose-degrading process. rRNA gene clone libraries for the samples collected in different stages of lignocellulose degradation process were constructed and analyzed. A total of 26 representative RFLP types were obtained from original soil clone library, including Mucoromycotina (29.5%), unclassified Zygomycetes (33.5%), Ascomycota (32.4%), and Basidiomycota (4.6%). When soil accumulated with natural lignocellulose, 16 RFLP types were identified from 8-day clone library, including Basidiomycota (62.5%), Ascomycota (36.1%), and Fungi incertae sedis (1.4%). After enrichment for 15 days, identified 11 RFLP types were placed in 3 fungal groups: Basidiomycota (86.9%), Ascomycota (11.5%), and Fungi incertae sedis (1.6%). The results showed richer, more diversity and abundance fungal groups in original forest soil. With the degradation of lignocellulose, fungal groups Mucoromycotina and Ascomycota decreased gradually, and wood-rotting fungi Basidiomycota increased and replaced the opportunist fungi to become predominant group. Most of the fungal clones identified in sample were related to the reported lignocellulose-decomposing strains. Understanding of the microbial community structure and dynamic change during natural lignocellulose-degrading process will provide us with an idea and a basis to construct available commercial lignocellulosic enzymes or microbial complex.

## 1. Introduction

Lignocellulose was extensively thought as a kind of promising cheap renewable resource for ethanol production. Especially considering limited fossil fuel crisis and controversial starch ethanol, using the lignocellulose residues as a raw material has become strong amazing and attracting [1–3]. However, the feasible technical route of bioethanol production from lignocellulose is in doubt. Its practical obstacles limit bioethanol production in a commercial scale. At present, the studies on ethanol production from lignocellulose mainly focus on three critical steps: pretreatment, enzymatic hydrolysis, and fermentation. The central question is the commercial technology for degrading lignocellulosic biomass to fermentable sugars. Particularly, the energy cost and the efficiency, become the crucial limitations of this process [2, 4].

In many natural habitats, lignocellulose degradation relies on complementary contribution of microbes. It carries out not only by pure culture of microorganism, but by a variety of lignocellulolytic species and some non-lignocellulolytic microbes to work synergically to break down the tough lignocellulosic structure [5–7]. Warnecke et al. use a metagenomic analysis of the bacterial community resident in the hindgut of a wood-feeding termite to reveal a broad diversity of bacteria and a large, diverse set of bacterial genes for cellulose and xylan hydrolysis. Many of these genes were expressed in vivo or had cellulase activity in vitro [8]. Hess et al. sequenced and identified 27,755 putative carbohydrate-active genes and expressed 90 candidate proteins from microbes adherent to plant fiber incubated in cow rumen, of which 57% were enzymatically active against cellulosic substrates [9]. So a promising way to break techniques obstacle in lignocellulose hydrolysis is to develop optimized enzyme or microorganism complexes [10–12]. However, we still know little about when these enzymes or microorganisms are involved in the process of lignocellulose degradation and how they functioned in dynamic change and succession regulations in different stages of wood biodegradation.

Forest soils contain huge natural pools of organic carbon compounds on the Earth, mainly composed by accumulating dead plant biomass on the forest floor. Organic matter decomposition by soil microorganisms in forest ecosystems plays a major part in the global C cycle. The understanding of organic matter decomposition in forest soil ecosystems is thus essential for any perspectives for developing available commercial microbial lignocellulose utilization strategies. This is special true for the tropical rain forest ecosystem. It displays high species diversity and complex community structure. For this ecosystem, environments keep in the orderly way throughout the year microorganisms make use of plant polysaccharides such as lignocellulose, starch, and protein to promote the rapid recycle of C, N source, and energy. In previous study, we investigate the microbial community structure and diversity in a rain forest soil. The results showed a variety of microbe related to the reported lignocellulose decomposing microorganisms, especially for a number of important wood-decaying fungi [13]. The subsequent characterization of cellulase and xylanase activities during a 50-day lignocellulose degrading process showed that the lignocellulolytic species complete a successive lignocellulose degrading process with an ordered spatial and temporal change [14]. In this study, fungal community structure and dynamic changes in different stages of forest soil lignocellulose degrading process were characterized and compared. Investigation of microbial composition, dynamic change, and succession regulations in natural lignocellulose biodegradation process will provide us with a basis to designate efficient enzymes or microorganisms complex in practice.

## 2. Materials and Methods

### 2.1. Sample Collection and Processing

The sample site was located in Xishuangbanna Tropical Botanical Garden, Yunnan, China (precipitation 1600 mm, mean annual temperature 21.4–22.6°C). Soil is latosol developed from Cretaceous sandstone [15]. There is 2-3 cm thick litter layer on the earth's surface. Basic physical and chemical properties of the soil are given in Table 1. The soil sample was collected from 5–8 cm soil layer and stored at 4°C until analysis.

To observe a successive degradation of lignocellulose, 10 g of dry soil was placed in 250 mL glass flasks. The soil was dispersed by adding 10 mL of deionized water and 7 g of natural lignocellulosic materials (wood sawdust : bagasse = 3 : 4). The glass flasks were incubated at 28°C.

### 2.2. PCR Amplification

Total DNA extraction was performed with Ultraclean Soil DNA Kit (MOBIO Laboratories, Inc., USA). A portion of 0.25 g of bulk soil sample was processed according to the protocol provided by the manufacturer. The quality of extracted DNA and approximate yields was determined by agarose gel electrophoresis. PCR amplification of fungal 18S rRNA genes from soil sample was carried out using the fungus-specific primer pair NSI (5′-GTA GTC ATA TGC TTG TCT C-3′) and FRI (5′-AIC CAT TCA ATC GGT AIT-3′) [16, 17]. Amplification was followed by the thermocycling pattern: 94°C for 3 min (1 cycle), 94°C for 30 s, 43°C for 30 s, 72°C for 90 s (30 cycles), and 72°C for 7 min (1 cycle). All PCR amplifications were carried out using a 2720 Thermal Cycler (Applied Biosystems, Gene Company Limited).

### 2.3. Cloning

PCR products were visualized on agarose gel stained with ethidium bromide. Bands were excised and DNA purified using an agarose gel DNA purification kit (Takara Bio Inc., Japan). Purified amplification products were cloned into pGEM T-easy vector system (Takara Bio Inc., Japan), and ligations were transformed into *Escherichia coli* DH-5*α* competent cells with ampicillin (100 *μ*g/mL) and blue/white screening in accordance with the manufacturer's directions.

### 2.4. RFLP Analysis

White clones were screened directly for inserts by performing colony PCR with vector primers M13-M3 and M13-RV. The amplifications were subjected to restriction fragment length polymorphism (RFLP) assay by enzymatic digestions with endonucleases TaqI, HaeIII, and HinfI following the manufacturer's instructions. And then, the digested DNA fragments were electrophoresed in 3% agarose gels. After staining with ethidium bromide, the gels were photographed and scanning image analyses were performed manually. Clone with unique restriction fragment length pattern (RFLP) was considered as a representative clone and sent for further sequence analysis.

### 2.5. Sequence Analysis

Sequences were checked for chimeric artifacts using the CHIMERA-CHECK program of the Ribosomal Database Project, RDP-II [18]. The resulting sequences (at least 700 bp) were compared with those available in NCBI using the BLAST search program and the RDP-II for fungi to determine their approximate phylogenetic affiliation and rRNA genes sequence similarities. Sequences differing only slightly (below 3%) were considered as a RFLP type, and each RFLP type was represented by a sequence [19]. Representative RFLP type sequence was aligned with fungal 18S rDNA sequences from NCBI and the RDP-II using ClustalX2.05 [20], and the alignment was corrected manually. Distance matrices and phylogenetic trees were calculated according to the Kimura 2-parameter model [21] and neighbor-joining [22] algorithms using the MEGA 5 software packages [23]. One thousand bootstraps were performed to assign confidence levels to the nodes in the trees.

### 2.6. Statistical Analysis

The RFLP data were used to estimate two diversity indices: the Shannon diversity index *H*′, a general diversity index, which considers both species richness and evenness [24]; and Pielou's evenness index *J*, uniformity of the distribution of individual [25] and coverage, the portion of the actual diversity that has been sampled [26].

### 2.7. Nucleotide Sequence Accession Numbers

The clones sequences determined in this study have been deposited in the GeneBank database under accession numbers GQ404733-GQ404785.

## 3. Results

### 3.1. Sample Characteristics and Processing

Soil samples BN-15 were collected from the hollow stump environment at Xishuangbanna Tropical Botanical Garden in Yunnan province of China. The results of lignocellulolytic capability and characteristics of the original soil and enriched samples are given in Table 1. With the development of lignocellulolytic process, both xylanase and cellulase activities gradually increased, and fiber content decreased from 17.13% to 12.57%. Xylanase activity increased from 513 U to 1258 U in primary 8 days, but the increasing became slow during later 7 days, only 812 U after a 15-day enrichment. However, cellulose activity of sample during enrichment increased by two times in primary 8 days and then sharply increased about four times in later 7 days, suggesting that in different stage there were different lignocellulose degradation content.

### 3.2. RFLP Analysis of 18S rRNA Gene

The total community DNA isolated from the original and enriched samples was of high molecular weight and sufficient purity for successful PCR amplification of fungal 18SrDNA gene. RFLP types were determined by observing *Hin*fI/HaeIII/HinfI digested colony PCR products. A total of 173 fungal clones from the original soil library, 72 clones from 8-day-enriched soil library, and 61 clones from 15-day-enriched soil library were analyzed. The clones with identical enzyme-digested patterns were put in the same RFLP group. Using this technique, the 173 uncultured clones were sorted into 26 distinct RFLP groups, the 72 clones cultured for 8 days were sorted into 16 distinct RFLP groups, and the 61 clones cultured for 15 days were classified into 11 different RFLP groups.

Coverage of three 18S rRNA gene clone libraries for original soil sample, 8-day enriched sample, and 15-day Enriched sample was separately 98.3%, 91.7%, and 93.4%, suggesting the number of analyzed clones is enough to reflect the community structure of the samples (Table 2). Comparison of Shannon *H*′ diversity values for the three sample showed that original soil samples had a higher microbial diversity and with the development of lignocellulolytic process, diversity of the fungal species decreased (Table 2).

### 3.3. Fungal Community and Their Dynamic Changes During Lignocellulolytic Process

**Figure 1:**
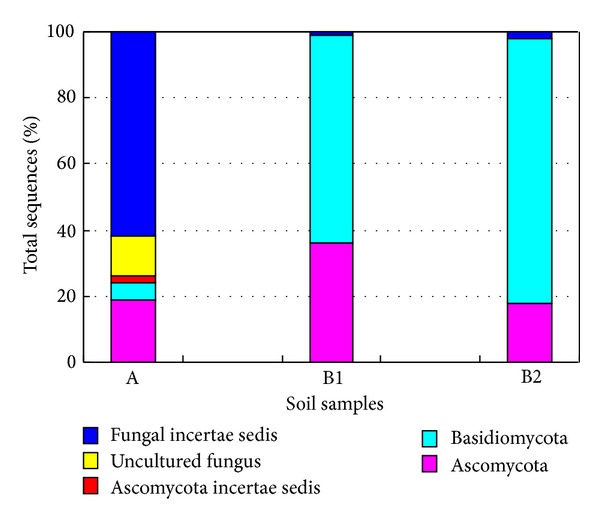
Comparison of fungal communities for original and enriched soil samples. An original soil sample from native rain forest; B1 enriched soil with natural lignocellulose biomass for 8 days; B2 enriched soil with natural lignocellulose biomass for 15 days.

One representative clone for each RFLP group was sequenced, and these sequences (approximately 750 bp) were searched for the organism with most similar sequences in NCBI nr database (Table 3). The RFLP sequence profiles revealed a pronounced shift in the relative abundance of the fungal populations during culturing with natural lignocellulosic biomass (8 and 15 days) (Figure 1). The 18S rDNA RFLP sequences of original soil are much richer, their diversity and abundance is higher than enriched soil samples. Contrarily, fungal populations profiles of 8 days and 15 days showed less diversity, and the dominance of few populations. And the dominant species in 8 days sample was also detectable in the 15 days, but it became less dominant.

**Figure 2:**
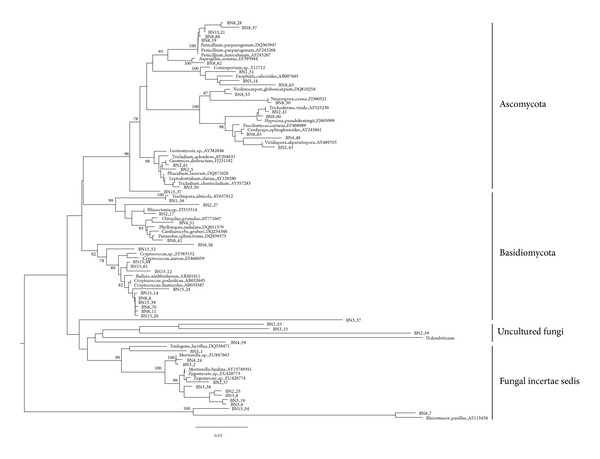
The phylogenetic relationships of fungal communities as compared to the most closely related sequences obtained from GenBank and RDP databases. The numbers at the nodes are the occurrence percentage with 1,000 bootstrap resamplings (values below 70% are not shown). The scale bar represents the number of changes per nucleotide position.

The RFLP types of original soil showed a rather high variability. A total of 26 representative RFLP types were obtained from 173 fungal clones for original soil clone library, including Fungi incertae sedis (9 RFLP types, 109 clones, 63.0%), Ascomycota (13 RFLP types, 56 clones, 32.4%), and Basidiomycota (4 RFLP types, 8 clones, 4.6%). Among them, fungal incertae sedis are divided into two categories: Mucoromycotina (5 RFLP types, 51 clones, 46.8%) and unclassified Zygomycetes (4 RFLP types, 58 clones, 53.2%) (Table 3, Figure 2). As shown in Shannon *H*′ diversity values, there are rather high diversity and abundant fungal groups in original forest soil, especially for Ascomycota, which included 13 different RFLP types from 56 clones, and Basidiomycota, 4 RFLP types from 8 clones. Ascomycota and Mucoromycotina were major predominant groups in the original rain forest soil. Among Ascomycota, uncultured Sarcosomataceae (2 RFLP type, 18 clones) was predominant species. And then, Leptodontidium elatius var. included 1 RFLP type, 15 clones. Most of fungal clones identified in the rain forest soil sample were related to members that have been reported to have highly lignocellulose decomposing strains and were extensively used in researches on related lignocellulose degrading genes and enzymes, or plant endophytes, or plant pathogens. Such as in Ascomycota, *Leptodontidium* is a microfungal endophytes in the root of plant and most *Sarcosomataceae* species are typically saprobic on rotten or buried wood [27]. *Penicillium* is high-efficiency strain of cellulose and *β*-glucosidase and had been applied by reconstructing [28]. *Phacidium lacerum*,* Exophiala calicioides*, and *Geomyces destructans* are all identified as plant root pathogens (Table 2) [27]. Most of RFLP types species belonging to Mucoromycotina were related to *Mortierella*, which is extensively studied as single-cell oil production fungi using lignocellulosic sugars [29]. Most of identified Basidiomycota clones belong to the wood rotting fungi, including *Clitopilus prunulus* (1 RFLP types, 2 clones), *Trechispora alnicola* (1 RFLP type, 2 clones), *Rhizoctonia *sp. CPCC 480725 (1 RFLP types, 2 clones), and *Phyllotopsis nidulans* (1 RFLP type, 2 clones) [6, 7].

To evaluate the microbial composition and dynamic changes in the lignocellulose degrading process, natural lignocellulosic materials were added into soil. With the successive degradation of lignocellulose, the diversity and number of fungal groups gradually reduced, especially for Ascomycota and Mucoromycotina and Basidiomycota is increasing and becoming the dominant group. In the soil accumulated for 8 days, 16 RFLP types were identified from 72 fungal clones, including Basidiomycota (5 RFLP types, 45 clones, 62.5%), Ascomycota (10 RFLP types, 26 clones, 36.1%), and Fungi incertae sedis (1 RFLP type, 1 clones, 1.4%) (Table 3, Figure 2). Ascomycota and Basidiomycota were absolutely predominant group. Ascomycota and Basidiomycota were absolutely predominant group; Ascomycota populations still kept higher diversity (10 RFLP types from26 clones). The Ascomycota identified in 8-day enriched sample mainly included *Aspergillus*, *Penicillium*, *Neurospora*, Hypocreales, *Neolinocarpon*, *Hypocrea*, and some unclassified fungi (Table 2, Figure 2). Among them, *Aspergillus*, *Penicillium* and *Neurospora* had been isolated and pure cultured. *Aspergillus* primarily produces pectinase and xylanase, which was widely used in cellulose decomposing [6, 7]. Except for *Aspergillus* and *Penicillium*, *Neurospora* is also excellent strain for producing cellulase and hemicellulase [30]. They are highly lignocellulose-producing strains for developing available industrial technologies and commercial enzyme products [31]. Basidiomycota mainly clustered into *Panaeolus*, *Cantharocybe*, *Clitopilus*, and a noncultivated Basidiomycota. *Panaeolus* is dominant Basidiomycota species, which is commonly used in cellulose decomposing [32]. For fungi incertae sedis, it just detected one clone; cluster analysis showed that it was closely related to *Rhizomucor*, which has been extensively reported to be high lignocellulose decomposing level [33].

But for the enriched samples of 15 days, 11 RFLP types identified from 61 clones were placed in 3 fungal groups: Basidiomycota (8 RFLP types, 53 clones, 86.9%), Ascomycota (2 RFLP types, 7 clones, 11.5%), and Fungi incertae sedis (1 RFLP type, 1 clones, 1.6%) (Table 3, Figure 2). Basidiomycota was dominant taxonomic group; most of Basidiomycota clustered with a yeast *Cryptococcus*, which commonly was found on leaves and made them decay [34]. Ascomycota were related to *Penicillium* and *Tricladium*, which usually were identified on the decaying wood in the nature [35]. Besides, fungi incertae sedis also clustered with *Rhizomucor*.

## 4. Discussion

Xishuangbanna tropical forest, characterized by its rapid recycle of carbon source, caught our primary interest as a system to understand microbial lignocellulose utilization strategies. There are much richer, diversity and abundance fungal groups in original forest soil. Fungal community in original soil is corresponding to the Fungi incertae sedis, including Mucoromycotina and unclassified Zygomycetes, followed by Ascomycota and Basidiomycota. Most of these fungi, including Ascomycota, Mucoromycotina, and Zygomycetes species are saprobes, ectomycorrhizal, or plant pathogens. To explore the changes of fungal structures and diversity in the process of lignocellulose degradation, we enriched and characterized the fungal consortia using the sugar cane bagasse and wood chips as natural carbon sources. The samples were collected in 8 days and 15 days, respectively. Through constructing 18S rRNA gene clone libraries and RFLP analysis, RFLP patterns showed that diversity and abundance of fungal community decreased with the development of lignocellulose degradation. The community structure was distinct in the different stages and so did for the predominant group. Most of fungal clones were related to members that have been reported to have highly lignocellulose decomposing strains and were extensively used in researches about related lignocellulose degrading genes and enzymes. Moreover, with the continuing degradation of lignocellulose, the diversity and number of Ascomycota gradually reduced in samples, but Basidiomycota increased, suggesting that their dominant group account for lignocellulose degrading changed and Basidiomycota could bring about a greater mass loss of litter. The result is consistent with previous observation of fungal species succession in woods using the microorganisms culturing method [36–38]. The fungal communities involved in lignocellulose degrading process achieved biodegradation of natural lignocellulose materials in an ordered shift and dynamic succession. Initially, some saprobes and opportunist, such as semiknown fungi, Zygomycota and Ascomycota, invade and account for advantage, which may be due to utilizing free organic matter. Along with the exhaustion of organic matter, fungal groups Mucoromycotina and Ascomycota decreased gradually and wood-rotting fungi such as Basidiomycota came to stage, which could break down the inner tough structure. Gradually, wood-rotting fungi replaced the opportunist fungi, and the process of decomposition is to enter stable period. In summary, the community structure in the different lignocellulose degrading stages is significantly distinct. Xishuangbanna tropical rain forest soil has its special and diverse lignocellulose degrading mechanism, possessing powerful ability to hydrolyze lignocellulose, thus promoting the rapid cycling of matter and energy.

High effective and economic utilization of biomass will have great influence on solving energy problems and facilitating social sustainable development. However, consuming a great deal of starch feedstock to produce biofuel will lead to world foodstuff crisis [39]. An alternative and effective resource for energy supplyment is the agricultural-derived lignocellulosic biomass, which is considered as potential material for future biomass to fue1 [40]. Present pretreatment process in conversion of biomass is energy consuming, expensive, and environment polluting. Understanding of the microbial community structure and dynamic change during natural lignocellulose degrading process will provide us with a basis to overcome the impediment. A dynamic lignocellulosic complex enzymes or microbes should be considered in the future designation.

## Figures and Tables

**Table 1:** Sample properties and their lignocellulose-degrading capabilities.

Soil sample	pH	MC (%)	Xylanase activity (U)	Cellulose activity (U)	Fiber content (g)
Original soil	5.38	31.1	342 ± 15	513 ± 18	0.1713
8-day enriched sample	6.12	41.2	756 ± 22	1258 ± 31	0.1602
15-day enriched sample	6.57	48.3	812 ± 26	4633 ± 29	0.1257

Mean values ± SE (*n* = 3).

**Table 2:** Estimates of coverage percent, Shannon diversity, and evenness index for BN-15 original and enriched soil samples.

	Number of clones	No. of RFLP types	Coverage %	Shannon index (*H*′)	Evenness index (*J*)
Original soil sample	173	26	98.3%	2.8073	0.8616
Enriched for 8 days	72	16	91.7%	2.1057	0.7595
Enriched for 15 days	61	11	93.4%	2.0859	0.8699

Data from both original soil clone and cultured soil clone libraries were shown. See text for the methods used to calculate these parameters.

**Table 3:** Most of fungal species in NCBI nr database for the 18S-rDNA sequences from the clone libraries^a^.

Samples	Accession number	RFLP type sequence	Clone numbers	Organism with most similar sequences in the NCBI databases	Similarity %	Taxon
Original soil	GQ404744	4–48	1	*Paecilomyces carneus*/EF468989	97%	*Ascomycota*-*Paecilomyces *
	GQ404745	2–43	2	*Viridispora diparietispora*/AY489703	99%	*Ascomycota*-*Viridispora *
	GQ404742	2–41	2	*Trichoderma viride*/AF525230	99%	*Ascomycota*-*Trichoderma *
	GQ404739	5–14	1	*Exophiala calicioides*/AB007685	97%	*Ascomycota*-*Exophiala *
	GQ404741	1–51	2	*Coniosporium* sp. CBS 665.80/Y11712	97%	*Ascomycota*-C*oniosporium *
	GQ404749	3–50	2	*Tricladium chaetocladium*/AY357283	99%	*Ascomycota*-*Tricladium *
	GQ404751	2–5	15	*Leptodontidium elatius var. elatius*/AY129280	99%	*Ascomycota*-*Leptodontidium *
	GQ404750	2–61	6	*Geomyces destructans*/FJ231102	99%	*Ascomycota*-*Geomyces *
	GQ404783	3–37	2	*Phacidium lacerum*/DQ471028	96%	*Ascomycota*-*Phacidium *
	GQ404754	1–55	4	Uncultured *Sarcosomataceae*/EF023269	91%	*Ascomycota*-*Sarcosomataceae *
	GQ404753	3–15	14	Uncultured *Sarcosomataceae*/EF023269	89%	*Ascomycota*-*Sarcosomataceae *
	GQ404755	2–59	2	*Leotiomycete* sp. G2-5 IC395/AY382646	85%	*Ascomycota*-*Leotiomycetes *
	GQ404765	4–59	3	Uncultured soil clone group I/EU179936	93%	*Ascomycota*-soil clone group I
	GQ404764	5–1	15	*Endogone lactiflua*/DQ536471	91%	Fungi incertae sedis-*Mucoromycotina*-*Endogone *
	GQ404757	3-2	1	*Mortierella* sp. A34/EU847663	99%	Fungi incertae sedis-*Mucoromycotina*-*Mortierella *
	GQ404756	4–24	4	*Mortierella alpine*/EU733605	99%	Fungi incertae sedis-*Mucoromycotina*-*Mortierella *
	GQ404758	3–8	21	*Zygomycete* sp. AM-2008a/EU428774	99%	Fungi incertae sedis-*unclassified Zygomycetes *
	GQ404759	2–25	12	*Zygomycete* sp. AM-2008a/EU428774	97%	Fungi incertae sedis-*unclassified Zygomycetes *
	GQ404763	2–57	5	*Zygomycete* sp. AM-2008a/EU428773	98%	Fungi incertae sedis-*unclassified Zygomycetes *
	GQ404760	3–19	20	*Zygomycete* sp. AM-2008a/EU428773	97%	Fungi incertae sedis-*unclassified Zygomycetes *
	GQ404762	3–38	21	*Mortierella hyaline*/AY157493	98%	Fungi ncertae sedis-*Mucoromycotina*-*Mortierella *
	GQ404761	5–6	10	*Mortierella hyaline*/AY157493	95%	Fungi incertae sedis-*Mucoromycotina*-*Mortierella *
	GQ404768	4–51	2	*Clitopilus prunulus*/AY771607	98%	*Basidiomycota*-*Clitopilus *
	GQ404769	2–27	2	*Phyllotopsis nidulans*/DQ851579	94%	*Basidiomycota*-*Phyllotopsis *
	GQ404770	2–17	2	*Rhizoctonia* sp. CPCC 480725/FJ515314	98%	*Basidiomycota*-*Rhizoctonia *
	GQ404771	1–59	2	*Trechispora alnicola*/AY657012	99%	*Basidiomycota*-*Trechispora *

8-day enriched sample	GQ404746	8–65	3	*Elaphocordyceps ophioglossoides*/AY245661	99%	*Ascomycota*-*Hypocreales *
	GQ404743	8–60	2	*Hypocrea pseudokoningii*/FJ605099	99%	*Ascomycota*-*Hypocrea *
	GQ404747	8–53	4	*Neolinocarpon globosicarpum*/DQ810258	99%	*Ascomycota*-*Neolinocarpon *
	GQ404748	8–50	6	*Neurospora crassa*/FJ360521	99%	*Ascomycot*a-*Neurospora *
	GQ404740	8–63	3	Uncultured fungus/EU733600	99%	Fungi
	GQ404738	8–62	3	*Aspergillus zonatus*/EF395944	98%	*Ascomycota*-*Aspergillus *
	GQ404737	8–19	2	*Penicillium purpurogenum*/AF245268	100%	*Ascomycota*-*Penicillium *
	GQ404736	8–66	1	*Penicillium purpurogenum*/DQ365947	99%	*Ascomycota*-*Penicillium *
	GQ404735	8–57	1	*Penicillium funiculosum*/AF245267	99%	*Ascomycota*-*Penicillium *
	GQ404734	8–28	1	*Penicillium purpurogenum*/DQ36547	99%	*Ascomycota*-*Penicillium *
	GQ404775	8–8	30	*Cantharocybe gruberi*/DQ234546	94%	*Basidiomycota*-*Cantharocybe *
	GQ404773	8–11	4	*Clitopilus prunulus*/AY771607	98%	*Basidiomycota*-*Clitopilus *
	GQ404767	8–41	1	*Panaeolus sphinctrinus*/DQ459375	98%	*Basidiomycota*-*Panaeolus *
	GQ404780	8–56	9	uncultured *Basidiomycota*/AF541994	94%	*Basidiomycota*-environmental samples
	GQ404774	8–70	1	*Asterotremella humicola*/AB035587	99%	*Basidiomycota*-*Asterotremella *
	GQ404784	8-7	1	*Rhizomucor pusillus*/AF113434	95%	Fungi incertae sedis-*Rhizomucor *

15-day enriched sample	GQ404782	15-14	3	*Cryptococcus podzolicus*/AB032645	99%	*Basidiomycota*-*Cryptococcus *
	GQ404733	15–21	1	*Penicillium funiculosum*/AF245267	99%	*Ascomycota*-*Penicillium *
	GQ404776	15–20	1	*Bullera ninhbinhensis*/AB261011	98%	*Basidiomycota*-B*ullera *
	GQ404752	15–37	6	*Tricladium splendens*/AY204631	96%	*Ascomycota*-*Tricladium *
	GQ404766	15–52	10	*Cryptococcus* sp. FYB-2007a/EF363152	96%	*Basidiomycota*-*Cryptococcus *
	GQ404778	15–44	11	*Cryptococcus aureus*/EF466059	98%	*Basidiomycota*-*Cryptococcus *
	GQ404781	15–12	8	*Cryptococcus* sp. FYB-2007a/EF363152	98%	*Basidiomycota*-*Cryptococcus *
	GQ404779	15–61	1	*Cryptococcus aureus*/EF466059	98%	*Basidiomycota*-*Cryptococcus *
	GQ404772	15–39	12	*Cryptococcus podzolicus*/AB032645	99%	*Basidiomycota*-*Cryptococcus *
	GQ404777	15–24	7	*Cryptococcus podzolicus*/AB032645	97%	*Basidiomycota*-*Cryptococcus *
	GQ404785	15–34	1	*Rhizomucor pusillus*/AF113434	94%	Fungi incertae sedis-*Rhizomucor *

^a^Sequences were compared to those in the NCBI database.
